# Supplementary material for: Mixture-of-Experts Variational Autoencoder for clustering and generating from similarity-based representations on single cell data
Source: PLoS Comput Biol. 2021 Jun 30;17(6):e1009086. doi: 10.1371/journal.pcbi.1009086 (PMC8277074; doi:10.1371/journal.pcbi.1009086)
Supplement: S1 Text — (ZIP) [file pcbi.1009086.s001.zip › S1_Text.pdf]

Supporting Information

# Mixture-of-Experts Variational Autoencoder for Clustering and Generating from Similarity-Based Representations on Single Cell Data

Andreas Kopf[1,4,5], Vincent Fortuin[2], Vignesh Ram Somnath[1], Manfred Claassen[3,4,*],

**1** Institute of Molecular Systems Biology, Department of Biology, ETH Zürich, Zurich, Switzerland.
**2** Biomedical Informatics Group, Department of Computer Science, ETH Zürich, Zurich, Switzerland.
**3** Division of Clinical Bioinformatics, Department of Internal Medicine I, University of Tübingen, Tübingen, Germany.
**4** Swiss Institute of Bioinformatics (SIB), Zurich, Switzerland.
**5** Life Science Graduate School Zurich, PhD Program Systems Biology, Zurich, Switzerland.

* manfred.claassen@med.uni-tuebingen.de

## 1 Metrics

### 1.1 F-measure

We compare results based on F-measure [1], which is defined as follows

$$F(C, K) = \sum_{c_i \in C} \frac{|c_i|}{N} \max_{k_j \in K} \{F(c_i, k_j)\} \tag{1}$$

where $N$ is the number of samples $C\{c_1, c_2, \ldots, c_n\}$ and $K\{k_1, k_2, \ldots, k_m\}$ are the cluster result and the reference cluster, respectively. Further $F(c_i, k_j)$ is the harmonic mean of precision and recall according to

$$F(c_i, k_j) = \frac{2Pr(c_i, k_j)Re(c_i, k_j)}{Pr(c_i, k_j) + Re(c_i, k_j)} \tag{2}$$

whereby $Pr(c_i, k_j)$ is the precision and $Re(c_i, k_j)$ is the recall.

### 1.2 Maximum Mean Discrepancy

One estimator of the Maximum Mean Discrepancy (MMD) [2] is defined as

$$\widehat{MMD}^2(X,Y) = t = \frac{1}{\binom{m}{2}} \sum_{i \neq i'} k(X_i, X_{i'}) + \frac{1}{\binom{m}{2}} \sum_{j \neq j'} k(Y_j, Y_{j'}) - \frac{2}{\binom{m}{2}} \sum_{i,j} k(X_i, Y_j) \tag{3}$$

where $X = \{\hat{x}_1, \cdots, \hat{x}_m\} \overset{iid}{\sim} P$, $Y = \{\hat{y}_1, \cdots, \hat{y}_m\} \overset{iid}{\sim} Q$ are samples from two distributions (e.g. samples from two different clusters of the latent representation, for MNIST of two different digits) and $k$ is a kernel function, where we use the popular RBF kernel. Based on that estimator Sutherland *et al.* [3] introduced the hypothesis test

$$H0 : P = Q \tag{4}$$

$$H1 : P \neq Q \tag{5}$$

using the statistic $\widehat{MMD}^2(X,Y)$. The distribution for $P$ and $Q$ is not required to be known. Sutherland *et al.* [3] used MMD and this test to train a Generative Adversarial Network (GAN) and also to evaluate the generative performance of the model. In this work we use $\widehat{MMD}^2(X,Y)$ to test if samples of different clusters of the latent representation are similar, or in other words the distance of the distributions. We used the Python implementation (`https://github.com/dougalsutherland/opt-mmd/blob/master/two_sample/mmd_test.py`) from Sutherland *et al.* [3].

# 2 Evaluation of MoE-Sim-VAE on synthetic data

We evaluated MoE-Sim-VAE on synthetic data sampled from a Gaussian mixture distribution with randomly sampled parameters. The Encoder and Decoder networks are dense layers stacked with depth parameters listed bellow. Further model and training details:

- number of experts: $\{2, \ldots, 40\}$
- batch size: 512
- code size: 10
- Number of iterations: 5000
- activation function; elu
- loss coefficient data reconstruction: 0.487
- loss coefficient clustering : 0.487
- loss coefficient mixture of Gaussian: 0.024
- learning rate: 0.001
- batch normalization

- dropout rate: 0.5
- distance threshold (perplexity parameter): 2
- encoder depth: 3
- encoder internal size: 50
- decoder depth: 3
- decoder internal size: 50
- depth clustering network: 5
- internal size clustering network: 100
- trainable parameters: depending on number of experts

# 3 Unsupervised clustering, representation learning and data generation on MNIST

We scaled the pixel values of the MNIST data between 0 and 1. In the following we define the architecture of MoE-Sim-VAE. The Encoder architecture:

- Convolution layer (filters 64, 3x3, elu activation)
- batch normalization
- Convolution layer (filters 128, 3x3, elu activation)
- batch normalization
- Max Pooling (2x2)
- batch normalization
- Convolution layer (filters 128, 3x3, elu activation)
- batch normalization
- Max Pooling (2x2)
- batch normalization
- Convolution layer (filters 64, 3x3, elu activation)
- Batch normalization
- Convolution layer (filters 8, 3x3, elu activation)
- Batch normalization
- Dense layer (code size)

Architecture of a Decoder expert:

- Dense layer (size=7x7x8)
- Convolution layer (filters 128, 3x3, elu activation)

- Upsampling layer (2x2)
- Convolution layer (filters 64, 3x3, elu activation)
- Upsampling layer (2x2)
- Convolution layer (filters 32, 3x3, elu activation)
- Convolution layer (filters 1, 3x3, elu activation)

Model and training details:

- number of experts: 10
- batch size: 128
- code size: 68
- Number of iterations: 20000
- activation function; elu
- loss coefficient data reconstruction: 0.487
- loss coefficient clustering : 0.487
- loss coefficient mixture of Gaussian: 0.024
- learning rate: 0.0001
- batch normalization
- dropout rate: 0.5
- k from kNN (perplexity parameter): 10
- depth clustering network: 3
- internal size clustering network: 200
- trainable parameters: 1619446

# 4 Clustering organ-specific single cell RNA-seq data

In all our experiments we filtered the data for genes with low total sum and low variance in expression. Therefore, we used the 20 percent lower quantile and ignored genes which fall underneath this threshold.

The competitor methods GMM, k-means, hierarchical, HDBSCAN, fuzzy-c-means and Louvain were applied on the first $k$ principal components of the scRNA-seq measurements. For a fair comparison we screened the number of principal components ($k \in [10, 20, \ldots, 100]$) and for Louvain clustering (resolution), HDBSCAN (minimum cluster size) and fuzzy-c-means (exponent for the fuzzy partition matrix) additional hyperparameter were tuned via grid search. HDBSCAN returns cells which are assigned to noise and not to one of the clusters. For fair comparison on the test dataset we assigned the noise cells to the nearest cluster centroid. All methods were applied on either principal componants of the counts itself or of Transcripts Per Million (TPM) normalized count data. Additionally, the methods where screened on 0-1

scaled data, which provided the better results compared to unscaled data. To generate scVI clustering results we followed steps in the scvi-tools documentation (`https://docs.scvi-tools.org/en/stable/user_guide/notebooks/api_overview.html`). We trained the scVI model only on the trainig data, but clustered on the whole dataset. Additionally, we grid-searched the resolution hyperparameter for Leiden-clustering. The best results based on F-measure, defined in Equation 1, were chosen.

The encoder and decoder networks are stacked dense layers with batch normalization and activation after each layer level. The clustering network is a stacked dense layer network as well, with activation in each dense layer and dropout afterwards. In the following we list the exact training- and model details:

- number of experts = 7
- batchsize = 256
- codesize = 20
- number of iterations = 20000
- activation fct = elu
- encoder depth = 1
- encoder internal size = 100
- decoder depth = 1
- decoder internal size = 100
- loss coef reconstruct data = 1
- loss coef KL divergence standard gaussian = 0.2
- loss coef clustering = 0.8
- learning rate = 0.001
- batch normalization = True
- dropout rate = 0.5
- depth cluster network = 1
- internal size cluster network = 100
- cluster loss coef kernel = 1
- cluster loss coef depict = 1
- cluster loss entropy = 1
- trainable parameters = 13694448
- best loss = 0.11028181

# 5 Learning cell type composition in peripheral blood mononuclear cells using CyTOF measurements

Weber and Robinson [4] provided preprocessed data online `https://github.com/lmweber/cytometry-clustering-comparison` for the datasets Levine_32dim, Levine_13dim, Samusik_01 and Samusik_all. For MoE-Sim-VAE, we additionally scaled the data between 0 and 1.

The raw Bodenmiller *et al.* [5] data can be downloaded from `http://www.cytobank.org/nolanlab/`. We normalized the raw data with an areakosinus hyperbolicus transformation

$$x_{norm} = arcsinh(\frac{x}{c}), \tag{6}$$

where the parameter $c = 5$ is chosen conventionally for CyTOF data [6]. Additionally for MoE-Sim-VAE we scaled the data between 0 and 1.

The Encoder and Decoder networks are three dense layers stacked with 20 units. Results are computed setting the loss coefficient for the KL loss of the VAE equal to zero, since we do not intend to generate any data, but rather give the chance to the AE to pick up the correct subpopulations. Further model and training details for all experiments on CyTOF data:

- number of experts: 25 [4], 15 [5]
- batch size: 128
- code size: 9
- Number of iterations: 30000 [4], 20000 [5]
- activation function: relu
- loss coefficient data reconstruction: 1
- loss coefficient clustering : 1
- loss coefficient mixture of Gaussian: 0
- learning rate: 0.001 [4], 0.005 [5]
- batch normalization
- dropout rate: 0.5
- distance threshold (perplexity parameter): 2
- distance metric: correlation
- depth clustering network: 5
- internal size clustering network: 9
- trainable parameters: 37563 [4], 22228 [5]

## References


1. Aghaeepour N., Finak G., FlowCAP Consortium, DREAM Consortium, Hoos H., Mosmann TR. *et al.* Critical assessment of automated flow cytometry data analysis techniques. Nature Methods, 2013.

2. Gretton A., Borgwardt K., Rasch M. J., Scholkopf B., Smola A. J. A Kernel Method for the Two-Sample Problem. arXiv, 2008.

3. Sutherland D. J., Tung H.-Y., Strathmann H., De S., Ramdas A., Smola A. *et al.* Generative models and model criticism via optimized maximum mean discrepancy. arXiv, 2019.

4. Weber L. M., Robinson M. D. Comparison of clustering methods for high-dimensional single-cell flow and mass cytometry data. Cytometry Part A, 2016.

5. Bodenmiller B., Zunder E. R., Finck R., Chen T. J., Savig E. S., Bruggner R. V. *et al.* Multiplexed mass cytometry profiling of cellular states perturbed by small-molecule regulators. Nature Biotechnology, 2012.

6. Galli, E., Hartmann, F.J., Schreiner, B., Ingelfinger F., Arvaniti E., Diebold M. *et al.* GM-CSF and CXCR4 define a T helper cell signature in multiple sclerosis. Nat Med 25, 1290–1300 (2019). https://doi.org/10.1038/s41591-019-0521-4
