# Supplementary material for: Mixture-of-Experts Variational Autoencoder for clustering and generating from similarity-based representations on single cell data
Source: PLoS Comput Biol. 2021 Jun 30;17(6):e1009086. doi: 10.1371/journal.pcbi.1009086 (PMC8277074; doi:10.1371/journal.pcbi.1009086)
Supplement: Attachment — Submitted filename: PCB-review-comments.pdf [file pcbi.1009086.s012.pdf]

Response to the referees' comments for manuscript PCOMPBIOL-D-20-02250

This is the response to the reviewer comments for the paper entitled "Mixture-of-Experts Variational Autoencoder for Clustering and Generating from Similarity-Based Representations on Single Cell Data". This work presents a representation learning approach to learn multi-modal distributions of high-dimensional data and use these to generate realistic synthetic data.

We thank the reviewers for their overall positive evaluation on novelty and significance of our contribution, as well as for their constructive suggestions. We have identified and addressed the following main issues raised by the reviewers:

1. **More detailed explanation of experimental details**: We added the requested information on the experiments
2. **Sensitivity of method performance with respect to selected hyperparameters:** We added additional experimental results assessing sensitivity, as well as discussion on the impact of hyperparameter selection.
3. **Referenced Github repository** for example code of MoE-Sim-VAE on clustering mouse organs based on scRNA-sequencing data.

Please see below a detailed response to the comments of both reviewers. Reviewer comments are displayed in blue font, author responses follow in normal font.

Reviewer #1:

The manuscript `Mixture-of-Experts Variational Autoencoder for Clustering and Generating from Similarity-Based Representations on Single Cell Data` proposes a generative clustering model, which is based on a variational autoencoder with a Gaussian mixture model for the latent space and a decoder consisting of a mixture of networks, where each mode in the latent space is decoded by an expert network. The gating network to assign samples to modes can be guided by prior knowledge on sample similarity. The authors demonstrate the performance of their model on MNIST data as well as single cell RNA-seq and mass cytometry clustering tasks. Overall, the work is interesting and the experiments conducted in a thorough manner. For an added value to the community, the authors should make their methods and scripts to reproduce the results accessible and provide additional details on their experiments. Other points are listed below.

Major points:
- Please make the code for the method and the scripts to reproduce the experiments accessible.
Example code of MoE-Sim-VAE is available on a Github repository (https://github.com/andkopf/MoESimVAE) for the example of clustering mouse organs on scRNA-seq data.

- What pre-processing was applied to the data presented in the Results? Please specify this and also provide details on the split of train and test data.
We added preprocessing of experiments to supplementary material. Specifically these comprise details on

- Clustering organ-specific single cell RNA-seq data
- MNIST, data scaling between 0-1
- Weber *et al.* provide the CyTOF data in preprocessed form Here we additionally scale between 0-1
- Bodenmiller *et al.* provided raw CyTOF data, which we preprocessed conventionally with a areakosinus hyperbolicus transformation and for optimization purposes scaled the data for MoE-Sim-VAE between 0-1

- - Several methods exist that use non-Gaussian variational distribution in a VAE, especially count based models in the single cell domain. These should be mentioned in p.2 l.14 and the results of a clustering in their latent space included in Table 2.
We added scVI clustering to Table 2. scVI is a well established modeling framework for single cell transcriptomic data and offers clustering on the latent representation as well as a scVI

representation to Figure 2 with true labels and clustered labels. Additionally, we added the exact experimental details to the supporting file.

- The ablation study nicely highlights the benefits of including a similarity matrix. However, the exact impact of the chosen similarity metric remains unclear. For this, a clustering based solely on the chosen similarity matrix should be included as a reference in all experimental results. Does the influence of the similarity matrix depend on the dimensions of the data or number of clusters? How sensitive is the method to misspecifications in the similarity (e.g. wrongly chosen signature genes in the cell type clustering problem)?
We added a table (Table S1) showing the exact accuracy of the selected signature gene similarity in the example of clustering mouse organs based on ssRNA-seq data to the supporting material and reported the average in the main text. For the remaining experiments we used a batch specific similarity measure based on a UMAP projection and the batch respective k-nearest neighbors which makes it difficult to compute a specific accuracy of the similarity. We nevertheless also showed for MNIST the significant impact of the choice of the similarity measure when comparing to a network trained without similarity clustering loss (Section: Unsupervised clustering, representation learning and data generation on MNIST and Figure S2). We further discussed the influence of the similarity matrix more in detail in the Discussion section.

Minor points:
- Could the authors comment on the motivation for the binary cross-entropy as reconstruction loss in Eq. (4). This seems to lead to blurry images in Fig. 2 and it is unclear why this should be a suitable loss for RNA-seq data.
MSE can be used, but previous work has shown that BCE works well for scRNA-seq data [Dongfang Wang and Jin Gu VASC: Dimension Reduction and Visualization of Single-cell RNA-seq Data by Deep Variational Autoencoder Genomics Proteomics Bioinformatics (2018)]. Additionally, it has been shown that BCE has better learning properties compared to MSE [Pavel Golik *et al.* Cross-entropy vs. squared error training: a theoretical and experimental comparison INTERSPEECH (2013)]. We discuss this issue in the revised manuscript (Section: Clustering organ-specific single cell RNA-seq data).

- What would be the guidelines to choose the number of clusters K in applications? This seems to be crucial for the ability to generate samples and according to Fig S1 many more experts than actual clusters in the data might be found.
We elaborate on this issue in more detail in the revised Discussion section. We showed that MoE-Sim-VAE did target less experts than defined on the example of synthetic data (Figure S1),

when clustering mouse-organs on scRNA-seq data (Figure 3) and when clustering cell types on mass cytometry measurements (Figure S8).

- Table 1 would be more insightful if the authors could provide a short description for these methods. Also, GANs for clustering seem an important alternative for the task but are not contained in the comparison nor mentioned in the text.
We added GAN performances on MNIST to Table 1.

- The ablation study could be included as part of Table 1.
We decided to abstain from including the ablation study in Table 1. since reporting a clustering performance without similarity loss is not meaningful, which in turn is required to train the network to cluster and gate the data. The purpose of the ablation study consists in showing that the latent representation is less sharp but still shows structure based on the reconstruction loss. Clustering results are at a F-measure of 0.18 but are not representative, since this is a random assignment due to the zero coefficient of the similarity clustering loss.

- l117-136 are hard to follow without going back to the original publication of VaDE.
We added related work section to paper for better explanation.

- The authors could remove repetitive parts of the text (e.g. method description of VaDE and MoE-Sim-VAE in Results, eq (2) and (3)) and better separate general concepts and technical details in the introduction.
To better structure and explain the competitor methods we introduced a Related work section in the manuscript.

- How sensitive is the method to the choice of pi_1 and pi_2?
We now elaborate on this issue in the revised Discussion section. Briefly, hyperparameter screening plays a crucial role, whereby a pattern was seen that reconstruction and clustering loss have higher coefficients compared to KL loss.

Reviewer #2:

The authors report a computational method, Mixture-of-Experts Similarity Variational Autoencoder, at clustering and data generation, with applications on large-scale single-cell data. The proposed mathematical framework is solid and builds upon ideas that are appropriate for the analysis of large-scale single-cell data. The authors have demonstrated the applicability of their method using publicly available data sets, and the figures and tables are simple and clear. I however have a few suggestions to improve the manuscript.

Major comments:
1. The authors do not a link to their implementation. This should be a red line for academic computational tools, and I would request the authors to share their implementation via a github repository reproducible in a revised version of the manuscript. In addition, the authors should include a vignette or a tutorial reproducing the results from at least one of the applications presented in the manuscript.
Example code of MoE-Sim-VAE is available on a Github repository (https://github.com/andkopf/MoESimVAE) for the example of clustering mouse organs on scRNA-seq data.

2. In Table 2 the authors extract the F-measure and NMI scores from a public review (Lukas et al. 2016). This is fine as long as the processing pipelines that the author's followed is the same as in the review. Minor changes in the data processing pipelines can lead to significant differences in the results. If this is the case, the authors should clarify. If not, the authors should verify the reported results with their own data processing pipelines.
We downloaded the processed data from the github repository and additionally scaled the features between 0 and 1 for MoE-Sim-VAE for optimization purposes.

Minor comments:
1. Fig3 is missing the colour legend
We added the legend.

2. Could the authors back the quantitative results shown in Fig4 with a visualisation of the data, with cells coloured by cluster / cellt ype?
We added Figure 8 to Supplementary data, which shows the reconstruction of the original data per expert in a PCA plot, for one example, since in total we computed MoE-Sim-VAE on nearby 270 different datasets in this section.

3. Why do the authors use the binary cross entropy as a reconstruction loss instead of the mean-squared error? Single-cell data is usually not scaled between 0 and 1.

MSE can be used, but previous work has shown that BCE works well for scRNA-seq data [Dongfang Wang and Jin Gu VASC: Dimension Reduction and Visualization of Single-cell RNA-seq Data by Deep Variational Autoencoder Genomics Proteomics Bioinformatics (2018)]. Additionally, it has been shown that BCE has better learning properties compared to MSE [Pavel Golik *et al.* Cross-entropy vs. squared error training: a theoretical and experimental comparison INTERSPEECH (2013)]. We discuss this issue in the revised manuscript (Section: Clustering organ-specific single cell RNA-seq data).
